# Wound Healing Potential of Topical Amlodipine in Full Thickness Wound of Rabbit

**DOI:** 10.17795/jjnpp-15638

**Published:** 2014-06-16

**Authors:** Ali Asghar Hemmati, Hoda Mojiri Forushani, Hossein Mohammad Asgari

**Affiliations:** 1 School of Pharmacy, The Physiology Research Center, Ahvaz Jundishapur University of Medical Sciences, Ahvaz, IR Iran; 2 School of Natural Resources, Khoramshahr University of Marine Science and Technology, Khoramshahr, IR Iran

**Keywords:** Wound Healing, Calcium Channel Blocker, Amlodipine, Phenytoin, Rabbits

## Abstract

**Background:**

Wound healing is a complicated and integrated process. Researches have indicated the wound healing effects of calcium channel blockers in animal models in recent years.

**Objectives:**

The aim of this study was to evaluate the wound-healing activity of amlodipine as a calcium channel blocker and combination of amlodipine with phenytoin on excisional cutaneous wound models in rabbit.

**Materials and Methods:**

Animals were divided into 5 groups (n = 5). The control group was treated topically with eucerin. The untreated control group received no healing agent. The reference standard group was treated with phenytoin1%. A treatment group was treated with amlodipine 1%. The last group was treated with combination of amlodipine1% and phenytoin 1%.

**Results:**

Results indicated significant difference between days needed for complete healing in both of the treatment groups. Wound closure was completed on 13th day and 9th day in amlodipine and combination groups respectively.

**Conclusions:**

In conclusion, calcium channel blockers can be used to enhance wound healing, especially if this treatment becomes with phenytoin. Further studies are needed to find out the mechanism of this healing effect.

## 1. Background

Wound healing represents a well-orchestrated reparative response, which occurs after all surgical procedures or traumatic injury. Wound healing is a complex multifactorial process, involving Inflammation, cell proliferation, angiogenesis, epithelialization, wound contraction, and matrix remodeling (1). This multifactorial sequence of processes starts from the moment of injury and continues for different periods varying based on the extent of injury and the health status of injured individual (2). Wound healing process is generally categorized into three integrated and overlapping phases as; the inflammatory phase, which is establishment of homeostasis and inflammation (3). During the inflammation phase, inflammatory cells are significantly increased in the wound site (1), which produce high amounts of reactive oxygen species (ROS) in wound tissue affecting wound healing (4). The proliferative phase, which is tissue granulation, contraction, and epithelialization, and the remodeling phase or resolution, which eventually determines the strength and appearance of the healed tissue (3). Studies have shown that several natural and synthetics products, promote the process of wound healing by influencing one or more phases of the healing process (5, 6). One such agent tried in wound healing is phenytoin. Phenytoin (diphenylhydantoin) was introduced first in 1937 for the effective control of convulsive disorders (7). A common side effect with phenytoin is gingival fibrous overgrowth (8). This apparent stimulatory effect of phenytoin on connective tissue suggested an exciting possibility for its use in wound healing (9). Previous clinical studies have shown that topical phenytoin promotes healing of ulcers (10). Calcium channel blockers (CCBs) have been used extensively in various cardiovascular conditions and may have a role in non-cardiac conditions as well (11). There are reports that cellular calcium metabolism appears to regulate extra cellular matrix and collagen production as well as wound healing (12, 13). It has been reported that antioxidants (vitamins A and E, Trolox) enhanced wound healing (14). Nifedipine and amlodipine by acting on voltage gated Ca^2+^ channels alter intracellular calcium and had antioxidant activity in some in vitro studies (15). Previous studies showed the potential of nifedipine to promote wound healing in human and animals (16).

## 2. Objectives

The present study was performed to assess the effect of amlodipine and combination of amlodipine and phenytoin on wound healing, using excision wound models in rabbits.

## 3. Materials and Methods

New Zealand rabbits of both sexes, weighing 1.4 to 1.9 kilograms were used. The animals were purchased from the Experimental Research Centre, Jundishapur University of Medical Sciences (Ahvaz, Iran). Before and after surgery, the animals were housed individually in cages (60 cm × 45 cm × 15 cm) and allowed to feed on a standard, commercial, pellet diet supplemented with fresh vegetables and tap water ad libitum. The test animals were kept in a holding room at a temperature of 22 ± 2°C and a humidity of 50 to 55 percent. Phenytoin (Daru Pakhsh, Iran) and amlodipine (Tehran Chemie, Iran) were used. A full thickness wound was made in the skin of test animals according to the method of Cross et al. Hairs of lower back and left flank of the test animals were fully shaved. The animals were held in standard crouching position. A template measuring 20 × 20 mm^2^ was placed on the stretched skin by using a fine tipped pen and this area was locally anaesthetized with a subcutaneous injection of lidocaine 2%. The wound was made by excising the skin, within the border of template to the level of loose subcutaneous tissue using a size 15 scalpel blade and a forceps (17). In this study, animals were divided into 5 groups (n = 5). Standard group was treated topically with eucerin containing phenytoin 1%. Control group received eucerin 1%. Untreated control group animals were not treated with any healing agent. A treatment group was treated with amlodipine 1% in eucerin. Another treatment group received combination of amlodipine 1% and phenytoin 1% in eucerin. All treatments were applied once a day until complete healing was attained. The animals were returned to the cages after treatment and their cages were changed daily kept clean to avoid wound infection. To determine wound healing, every 24 hours each test animal was held in the standard crouching position and the outline of the wound was traced on a transparent plastic sheet using a fine tipped pen. Measurement errors were minimized by repeating each measurement three times and using the average of measurements in all calculations.

### 3.1. Statistical Analysis

The area of the wounds on the first day was considered as 100 percent and the wound areas on subsequent days were compared with the wound on the first day. Healing percentage was the difference between the initial wound and the healing wound. Data was analyzed using SPSS version (13). All the results were expressed as mean ± SD. Unpaired t-test was used to compare area of wound healing with control group. Unpaired t-test was used to compare time required for the complete wound closure between the groups. P < 0.05 was considered to be significant.

## 4. Results

**Figure 1.**
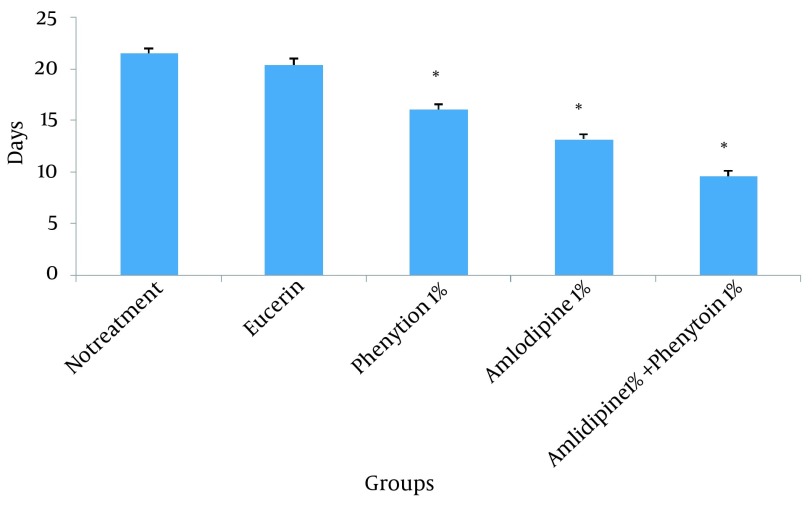
Time (days) Required for Complete Wound Healing After Application of Tested Compounds Significant difference from control group is shown * (P < 0.001).

**Figure 2.**
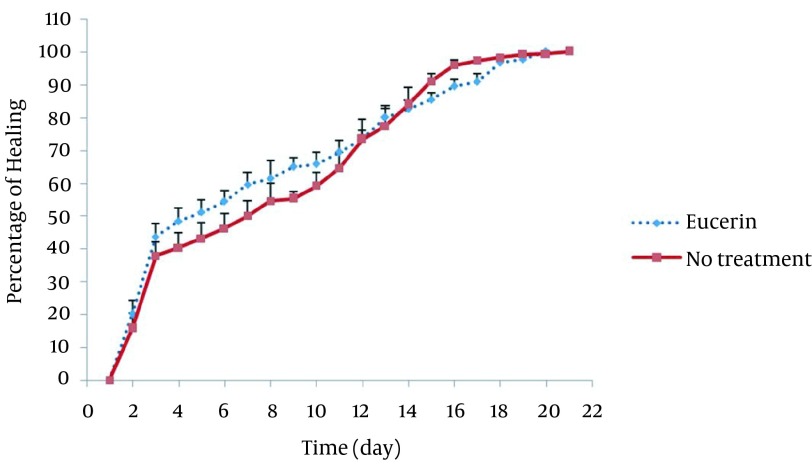
Wound Healing Profile of Non-Treated and Eucerin-Treated Groups No significant difference was seen between the two groups.

**Figure 3.**
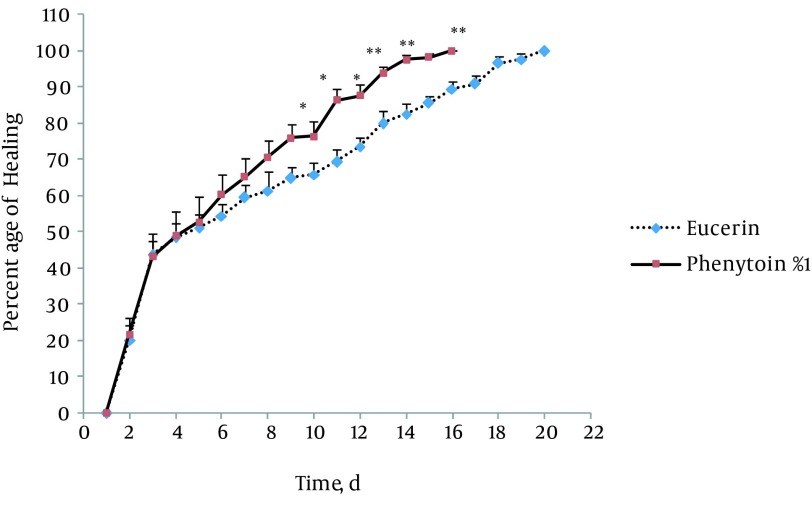
Wound Healing Profile of Eucerin-Treated and Phenytoin 1% Treated Groups Significant difference was seen from 10th day. Healing completed on 16th day in eucerin-treated group. * P < 0.05; ** P < 0.01 indicates significant difference from control group.

**Figure 4.**
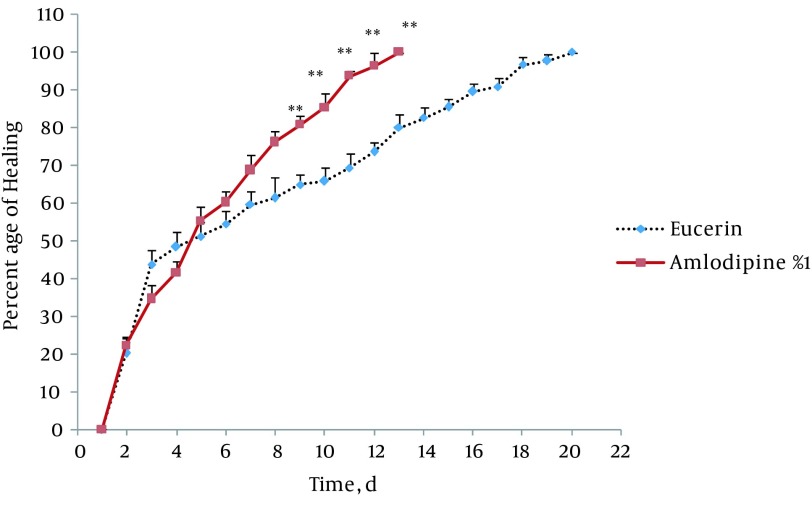
Wound Healing Profile of Eucerin-Treated and Amlodipine 1%-Treated Groups Significant difference was seen from 9th day. Healing completed on day 13 in amlodipine1%-treated group. ** P < 0.01 indicates significant difference from control group.

**Figure 5.**
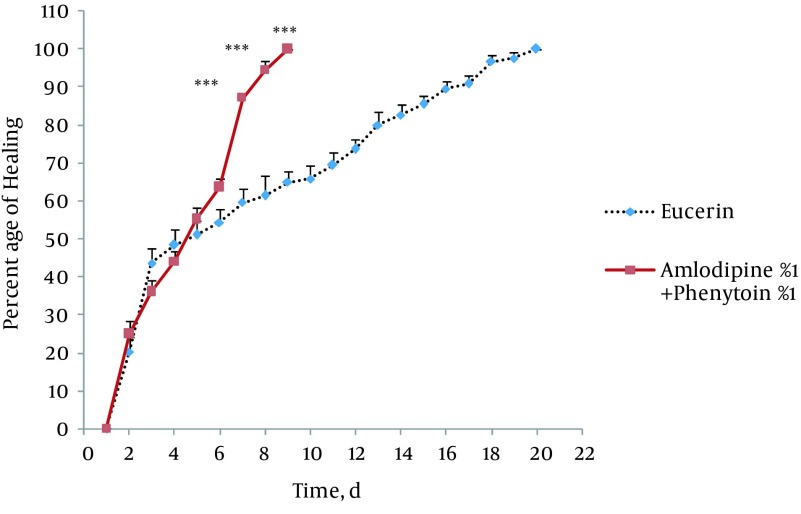
Wound Healing Profile of Eucerin-Treated and Amlodipine 1% + Phenytoin 1%-Treated Groups Significant difference was seen from the seventh day. Healing completed on ninth day in amlodipine + phenytoin-treated group. Significant different from control group was shown *** (P < 0.001).

In non-treated group, wound healing completed within 21 days and 20 days in the group treated with eucerin. Wound healing in animals treated with eucerin containing phenytoin 1% required 16 days. Treatment groups with eucerin containing amlodipine 1% and amlodipine 1% + phenytoin 1% completed within 13 and 9 days, respectively (Figure 1). Treatment of test animals with eucerin had no significant improvement as compared with non-treated control animals (Figure 2). Phenytoin caused complete healing in 16th day compared to 21th day for eucerin group (Figure 3). The healing effect of amlodipine 1% has been shown in Figure 4. Wound healing was 100 percent in the amlodipine 1% group on 13th day of treatment. Figure 5 shows that significant differences between eucerin and combination of phenytoin 1% and amlodipine 1% group were observed seven days after the initiation of treatment, which completed after 9 days.

## 5. Discussion

Phenytoin is one of the most commonly prescribed medications to treat epilepsy and may also be used in cases of neuralgias and cardiac arrhythmias (18). It is estimated that about 30% to 50% of patients taking phenytoin develop significant gingival alterations (19). Microscopic analysis of Phenytoin-induced gingival overgrowth biopsies revealed a redundant tissue of apparently regular composition or with an increased amount of collagen and number of fibroblasts (20). Inhibition of synthesis or secretion of collagenase by fibroblasts is a suggested mechanism of phenytoin in wound healing (21). Amlodipine is a long-acting calcium channel blocker belonging to dihydropyridine group, which is used for the treatment of hypertension and angina (22). Side effects of amlodipine usage involve headache, dizziness, edema, flushing, palpitation and rarely gingival hyperplasia (23). Amlodipine-associated gingival hyperplasia was first reported in 1993 (23). Gingival hyperplasia due to the use of amlodipine was found in 5 (3.3%) of 150 patients in the study performed by Jorgensen et al. (24) and in three (1.7%) of 181 patients in the study conducted by Ellis et al. (25) and in 4 (1.3%) of 301 patients in the study performed by Ono et al. (26). The reasons for choosing amlodipine in this study were:

- Other calcium channel blockers were used as healing agents in previous studies, but amlodipine was not (16, 27).
- Amlodipine is safer with less adverse effects and more availability among other calcium channel blockers (22, 28).

Some studies showed an association between the use of CCBs and gingival hyperplasia (29, 30). Two main inflammatory and non-inflammatory pathways have been already suggested. The proposed non-inflammatory mechanisms include defective collagenase activity due to decreased uptake of folic acid. In the inflammatory pathway, the inflammation could lead to upregulation of several cytokine factors such as TGFß1 (31). Therefore, the wound healing effect of CCBs may be related to stimulation of growth factors. The presence of growth factors is necessary in wound healing process. On the other hand, CCBs are known to cause vasodilatation, which increases the blood supply to injured region. Hence, these CCBs could be used safely in patients undergoing surgery since they do not adversely affect healing (28).

Wound healing potency of new calcium channel blockers is subject of recent researches. One study recently showed that azelnidipine (AZL), a new calcium channel blocker with antioxidant properties, would enhance wound healing in streptozotocin-induced diabetic rats by restoring NO synthesis (27). In this study, amlodipine significantly enhanced wound healing in rabbit and decreased the days needed for complete healing compared to control group. This survey indicated that combination of phenytoin and amlodipine is more effective than amlodipine and phenytoin alone in healing of skin wound in rabbit. It seems that using combination of two different medical groups, which act presumably by different mechanisms decrease the days of healing as an advantage of this investigation. From the clinical point of view, administration of such compounds may help to reduce the cost of treatment and provide more comfort for patients with acute or chronic wounds. However, further investigations are required to elucidate the mechanisms involved in healing property of this combination or amlodipine alone.
